# An interspecies comparative study of invasive electrophysiological functional connectivity

**DOI:** 10.1002/brb3.863

**Published:** 2017-11-22

**Authors:** Kaitlyn Casimo, Lila H. Levinson, Stavros Zanos, C. Alexis Gkogkidis, Tonio Ball, Eberhard Fetz, Kurt E. Weaver, Jeffrey G. Ojemann

**Affiliations:** ^1^ Graduate Program in Neuroscience University of Washington Seattle WA USA; ^2^ Center for Sensorimotor Neural Engineering University of Washington Seattle WA USA; ^3^ Wesleyan University Middletown CT USA; ^4^ Department of Physiology and Biophysics University of Washington Seattle WA USA; ^5^ Washington National Primate Research Center University of Washington Seattle WA USA; ^6^ Feinstein Institute for Medical Research New York City NY USA; ^7^ Translational Neurotechnology Laboratory Department of Neurosurgery Faculty of Medicine Medical Center – University of Freiburg Freiburg Germany; ^8^ Laboratory for Biomedical Microtechnology Department of Microsystems Engineering Faculty of Engineering University of Freiburg Freiburg Germany; ^9^ Department of Radiology University of Washington Seattle WA USA; ^10^ Integrated Brain Imaging Center University of Washington Seattle WA USA; ^11^ Department of Neurological Surgery University of Washington Seattle WA USA; ^12^ Department of Neurological Surgery Seattle Children's Hospital Seattle WA USA

**Keywords:** connectivity, electrocorticography, electrophysiology, functional connectivity, macaque, sheep, synchrony

## Abstract

**Introduction:**

Resting‐state connectivity patterns have been observed in humans and other mammal species, and can be recorded using a variety of different technologies. Functional connectivity has been previously compared between species using resting‐state fMRI, but not in electrophysiological studies.

**Methods:**

We compared connectivity with implanted electrodes in humans (electrocorticography) to macaques and sheep (microelectrocorticography), which are capable of recording neural data at high frequencies with spatial precision. We specifically examined synchrony, implicated in functional integration between regions.

**Results:**

We found that connectivity strength was overwhelmingly similar in humans and monkeys for pairs of two different brain regions (prefrontal, motor, premotor, parietal), but differed more often within single brain regions. The two connectivity measures, correlation and phase locking value, were similar in most comparisons. Connectivity strength agreed more often between the species at higher frequencies. Where the species differed, monkey synchrony was stronger than human in all but one case. In contrast, human and sheep connectivity within somatosensory cortex diverged in almost all frequencies, with human connectivity stronger than sheep.

**Discussion:**

Our findings imply greater heterogeneity within regions in humans than in monkeys, but comparable functional interactions between regions in the two species. This suggests that monkeys may be effectively used to probe resting‐state connectivity in humans, and that such findings can then be validated in humans. Although the discrepancy between humans and sheep is larger, we suggest that findings from sheep in highly invasive studies may be used to provide guidance for studies in other species.

## 1 INTRODUCTION

Functional connectivity (FC) tracks dynamic, time‐varying statistical interactions reflective of structured multisynaptic connections, and is believed to indicate transient interactions between neuroanatomical hubs (Schölvinck, Leopold, Brookes, & Khader, 2013). fMRI estimates of FC have proven particularly popular, due to the completely noninvasive nature of the technology. Many fMRI studies comparing connectivity across species have done so in the resting state, a period devoid of structured behavioral demands (Fox & Raichle, 2007; Zhang et al., 2010). Spontaneous, behaviorally independent FC generally conforms to an underlying anatomical framework and varies as a function of time (Damoiseaux & Greicius, 2009; Deco, Jirsa, & McIntosh, 2011). However, fMRI is limited in the temporal domain to oscillatory interactions occurring on the order of 0.01 Hz (Fox & Raichle, 2007).

In contrast, electrophysiological measurements of FC can resolve these oscillatory dynamics at millisecond time scale (Schölvinck et al., 2013). Neurophysiological studies on these oscillations have highlighted how canonical frequency band dynamics reflect different levels and modes of neural connectivity, such as thalamocortical and cortico‐cortical interactions (Wang,^2010^). Our examination of connectivity specifically in resting state enables comparison between species, independent of cross‐species behavioral or performance discrepancies (Goulas et al., 2014; He, Snyder, Zempel, Smyth, & Raichle, 2008). In addition to the obvious relevance of nonhuman primate research to human neurophysiology, nonhuman primates and other large animal models are frequently used as analogs for humans, especially for electrode development (Gierthmuehlen et al., 2014; Kohler et al., 2017).

Here, we aim to characterize and compare properties of resting‐state connectivity in the human and macaque brain using invasive electrophysiology, supporting our findings with comparable data from sheep. Our primary focus is comparison of human and macaque monkey connectivity between brain regions.

### 1.1 Comparative anatomy between humans and nonhuman primates

The brains of nonhuman primates resemble those of humans in cytoarchitecture (Hackett, Preuss, & Kaas, 2001; Petrides & Pandya, 1999) and functional organization (Koyama et al., 2004; Rees, Friston, & Koch, 2000). Nonhuman primates and humans share similarities in cortical neuronal density and projection patterns, both between layers and between different regions. Humans and nonhuman primates are broadly more similar in the relative size of various functional brain regions than humans are to more distantly related species (Krubitzer, 2007). Divergence is more in the relative sizes of the regions (Hackett et al., 2001; Krubitzer, 2007; Petrides & Pandya, 1999), in particular in the prefrontal cortex (Saleem, Miller, & Price, 2014).

For example, comparison of macaque and human visual system fMRI data reveals that relative to macaques, the human parietal, temporal, and frontal cortices have functionally expanded more than the occipital cortex (Buckner & Krienen, 2013; Orban, Van Essen, & Vanduffel, 2004). Overall, though, the relative level of similarity between macaque and human brains makes them a particularly useful model organism for comparative study.

### 1.2 Comparative functional connectivity

Macaques have often been compared directly to humans in both task‐based (Grefkes, Weiss, Zilles, & Fink, 2002; Joly et al., 2012) and resting state‐(Hutchison, Womelsdorf, Gati, Everling, & Menon, 2013; Mantini, Perrucci, Del Gratta, Romani, & Corbetta, 2007; Margulies et al., 2009) fMRI studies. These extensive studies have broadly found that the species share many similarities in connectivity, especially interregional connectivity (Hutchison et al., 2012; Hutchison et al., 2013; Margulies et al., 2009; Zhang et al., 2010). Patterned resting‐state connectivity has been observed in resting fMRI data in such diverse species as mice (Sforazzini, Schwarz, Galbusera, Bifone, & Gozzi, 2014), rats (Liang, King, & Zhang, 2011; Zhang et al., 2010), macaques (Vincent et al., 2007), and chimpanzees (Rilling et al., 2007). Monkey species in particular, with their extensive homology to humans, offer specific opportunities for studies that contribute to understanding of human resting‐state function (Hutchison & Everling, 2012).

Given the inherent challenges in comparing performance differences between species, investigating functional connectivity patterns in the resting state provides distinct advantages (reviewed in Hutchison & Everling, 2012). However, a true resting state is difficult to induce and characterize in animals without the use of sedation, as animals cannot be instructed to be specifically inactive. Anesthesia has been found to be only roughly equivalent to resting state in humans (Breshears et al., 2012), pigs (Tanosaki, Ishibashi, Zhang, & Okada, 2014), macaques and other monkey species (Vincent et al., 2007), and mice (Grandjean, Schroeter, Batata, & Rudin, 2014). Our understanding of differences in connectivity under anesthesia relative to waking state remains incomplete, and limits the application of animal anesthesia studies to understand human resting state. Here, we circumvent this common limitation by only using waking humans and animals, and specifically delineating resting state in our animals through video monitoring.

While intracranial electrodes have long been used in monkey studies, including specifically for resting‐state connectivity (e.g., Fukushima, Saunders, Leopold, Mishkin, & Averbeck, 2012), they have not to our knowledge been used for direct comparison to human resting‐state connectivity. Monkey functional (Belcher et al., 2013) and structural (Goulas et al., 2014) connectivity is observed to be globally similar to humans, but more dissimilar locally.

### 1.3 Advantages of electrophysiology for comparative investigation

Although resting‐state fMRI remains the gold standard for functional connectivity studies, a number of potential confounds, including nonneuronal noise and artifacts introduced by motion, may impact inferences drawn from comparative studies (reviewed in Murphy, Birn, & Bandettini, 2013). More importantly, the hemodynamic BOLD signal is an indirect measure of neural activity and the cascade of physiological events linking neural activity to hemodynamics may not be homologous across species, as it heavily relies on glial function, which has tremendous cross‐species variability (Oberheim et al., 2009). Electrophysiological methods, particularly invasive direct cortical recording with electrocorticography (ECoG), bypass some of these issues, though they have limits of their own.

ECoG monitoring for seizure localization in patients with intractable epilepsy provides a unique opportunity to record electrophysiological data directly from the human cortex, producing robust differentiation of all major canonical frequency bands. This oscillatory activity is far beyond the temporal resolution of fMRI. The high frequencies of the cortical spectrum (high gamma [HG], 70–200 Hz), a range that is thought to best reflect local cortical computation (Miller, Sorensen, Ojemann, & den Nijs, 2009), are particularly challenging to capture clearly from outside the skull using surface recording technology such as EEG and MEG (Crone, Sinai, & Korzeniewska, 2006). Low signal‐to‐noise ratio (Crone, Miglioretti, Gordon, & Lesser, 1998) and high spatial and temporal resolution make ECoG an especially valuable tool for connectivity analysis, especially for synchrony measures such as those we use here, as timing of the neural signals is captured with high fidelity by ECoG. However, the invasive nature of ECoG surgery translates to few patients and limited time to perform research, and each patient only has electrode coverage over a portion of the brain. Similar electrophysiological data from other animals, combined with data from experiments that can be performed in animals but not in humans, provide support for ECoG findings in humans.

Resting‐state connectivity has been successfully characterized in humans from multiple band‐limited frequencies of ECoG data using a variety of measures (Casimo et al., 2016; Ko, Darvas, Poliakov, Ojemann, & Sorensen, 2011; Weaver et al., 2016) including coherence, amplitude correlation, phase locking, and various cross‐frequency coupling approaches (Foster, Rangarajan, Shirer, & Parvizi, 2015; Schölvinck et al., 2013). Recent work has indicated correspondence between electrophysiological properties recorded with ECoG, especially in 0.01–0.1 Hz modulations of the HG band, correlate well with BOLD signals, indicating spatial consistency and a possible physiological link between the phenomena (Keller et al., 2013; Ko, Weaver, Hakimian, & Ojemann, 2013; Ko et al., 2011). Although we are exclusively examining electrophysiological data here, this prior work indicates some degree of transferability to similar fMRI comparative studies exists.

ECoG has been used with nonhuman primates for large‐scale connectivity studies (Hutchison & Everling, 2012; Liu, Yanagawa, Leopold, Fujii, & Duyn, 2015; Vincent et al., 2007; Wu et al., 2016; Yanagawa, Chao, Hasegawa, & Fujii, 2013). However, to our knowledge, no direct comparison between humans’ and nonhuman primates’ resting electrophysiological connectivity has been conducted.

Sheep are also strong candidates for chronic implantation of ECoG grids, as unlike mice and rats, they have large enough cranial capacity for approximately the same size ECoG grids used in human experiments (Gierthmuehlen et al., 2014), which limits variation resulting from recording modality. Although sheep have not to our knowledge been evaluated for resting‐state connectivity in ECoG or any other modality, somatosensory‐evoked potentials have been successfully recorded in sheep implanted chronically with a micro‐ECoG grid (Gierthmuehlen et al., 2014). These grids, also used here, have smaller electrode diameters and decreased interelectrode spacing than typical macroscale ECoG grids used in invasive human epilepsy studies. Although this provides higher spatial resolution with less coverage, micro‐ECoG is still sensitive to population‐scale potentials.

This study compares electrophysiological resting‐state connectivity using invasive ECoG recordings in rhesus macaque monkeys and humans, extending previous comparisons (Hutchison, Womelsdorf, Gati et al., 2013; Hutchison, Womelsdorf, Allen, et al., 2013; Mantini et al., 2011; Margulies et al., 2009; Vincent, Kahn, Snyder, Raichle, & Buckner, 2008) in the fMRI literature (reviewed in Hutchison & Everling, 2012). We focus on connectivity within and between pairs of homologous brain regions in humans and macaque monkeys. We supplement our findings in monkeys with additional data from somatosensory cortex in sheep.

## 2 METHODS

### 2.1 Animal and human subjects

This study includes data from humans, macaque monkeys, and sheep. Age and sex data on all subjects are described in Table 1. All experiments described here were approved by the appropriate ethics boards: University of Washington IRB (humans), University of Washington IACUC (monkeys), Animal Committee of the University of Freiburg, Regierungspräsidium Freiburg, Baden‐Wuerttemberg, and EU directive 2010/63/EU (sheep).

**Table 1:** Characteristics and electrodes for all subjects

	Age	Sex	Electrodes (included, per area)
Human 1	43	M	108 (PFC: 31; M1: 2; PM: 10; PC: 3)
Human 2	44	M	90 (PFC: 8; M1: 1; PM: 12; PC: 5)
Human 3	20	M	64 (PFC: 0; M1: 0; PM: 0; PC: 31)
Human 4	31	F	82 (PFC: 9; M1: 1; PM: 12; PC: 9)
Monkey 1	6	M	32 (PFC: 3; PM: 6; M1: 18)
Monkey 2	5	M	20 (PFC: 4, M1: 11; PC: 2)
Monkey 3	4	M	35 (PFC: 9; PM: 10; M1: 1; PC: 8)
Sheep 1	Adult	M	16 (S1: 16)
Sheep 2	Adult	F	16 (S1: 16)

### 2.2 Electrode implants and data collection

**Figure 1:**
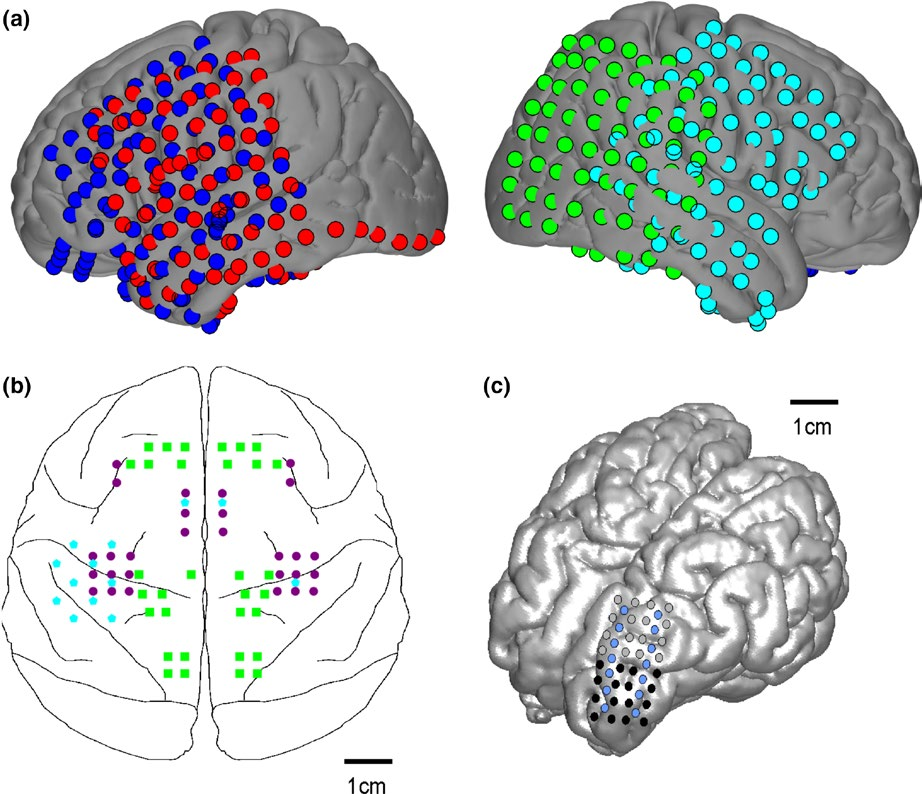
Locations of electrodes in human (a), macaque monkey (b), and sheep (c) subjects. Humans and monkeys are color coded by subject. Sheep electrodes are in the same locations for both sheep; black is recording, blue is reference, gray is unused

Human subjects (Table 1) were undergoing long‐term electrocorticographic (ECoG) monitoring with video for epilepsy at Harborview Medical Center in Seattle, WA. Patients were implanted with subdural platinum ECoG arrays (Ad‐Tech, Racine, WI, or Integra Lifesciences, Plainsboro, NJ) for the clinical purpose of localizing medically intractable epilepsy. Electrodes were placed in grid and strip configurations (Figure 1a), with a scalp reference, 2.3‐mm electrode surface diameter, and 1 cm interelectrode spacing. Electrode locations in human subjects were based entirely on clinical indications. From a group of nine ECoG patients who consented to research, electrode placement from four subjects overlapped with homologous, gross cortical structures covered by the aggregate of the monkeys’ electrode placement (Figure 1b). Time series data were extracted from these four patients’ clinical recordings taken with standard clinical epilepsy monitoring equipment (Xltek EEG Systems, Natus Medical Incorporated) sampled at 1000 Hz.

Resting‐state electrophysiological recordings and accompanying video were obtained from three rhesus macaque monkeys (Table 1), which were housed at the Washington National Primate Research Center (RRID:SCR_002761) and were part of other ongoing research. Monkeys were implanted with micro‐ECoG electrodes (custom‐made; 0.2 mm diameter) (Figure 1b) and a distant skull screw ground. Monkey implant locations were determined based on the needs of other studies. Electrophysiological data were recorded with a Tucker–Davis Technologies system (Alachua, FL) at a sampling rate of 1200 Hz. Penetrating electrode arrays present in two of the three monkeys were excluded from analysis.

Sheep subjects (Table 1) were implanted with chronic, wireless implants (CorTec GmbH, Freiburg, Germany) consisting of an intracorporeal processing unit, a custom micro‐ECoG electrode array (0.8 mm contact diameter, 4‐mm intercontact spacing, 16 recording contacts) and an extracorporeal unit for communication with the implant (Kohler et al., 2017). Chronic implantations were performed for the purpose of testing electrode design and long‐term performance. The micro‐ECoG electrode arrays were placed over the somatosensory cortex and adjacent sensory areas (Figure 1c), which were localized by landmarks derived from prior studies using somatosensory‐evoked potentials (Gierthmuehlen et al., 2014) and anatomy (Dinopoulos, Karamanlidis, Papadopoulos, Antonopoulos, & Michaloudi, 1985; Johnson, Rubel, & Hatton, 1974). During resting state (unmoving, unrestrained), data were recorded at a sampling frequency of 1000 Hz.

In all three species, video recordings (humans, monkeys) or live observations (sheep) were used to confirm that subjects were resting for an uninterrupted period with their eyes open or mostly open. In both humans and monkeys, three 10‐minute periods in which the subject was quiet, unmoving, and awake were identified and labeled as rest. In sheep, equivalent 3‐minute periods were identified. The identified time segments were then extracted from the larger ECoG data streams.

### 2.3 Data analysis

#### 2.3.1 Data processing

Data preprocessing for all species was conducted in MATLAB (MathWorks, Natick, MA; RRID:SCR_001622) computing environment as described in Casimo et al. (2016). This included common average re‐referencing, notch filtering to remove line noise (humans, monkeys: 60 Hz and harmonics; sheep: 50 Hz and harmonics), and exclusion of interictal artifacts in human subjects using clinical labels. Human and monkey recordings were resampled to 600 Hz (Casimo et al., 2016). For all recordings, amplitude and phase angle were extracted using a nonanalytic Morlet wavelet with ¼ octave resolution for pseudofrequencies 1–200 Hz. We then averaged across the phases and amplitudes for the frequency bins that fell within each canonical frequency band of interest (delta, 0–4 Hz; theta, 4–8 Hz; alpha, 8–12 Hz; beta, 13–30 Hz; gamma, 30–70 Hz; HG, 70–200 Hz).

Anatomical locations of humans’ electrode locations were identified as previously described (Casimo et al., 2016). We aligned and registered each subject's preoperative MRI with a postoperative CT indicating electrode position using BioImage Suite (Papademetris et al., 2006; RRID:SCR_002986). We then registered each native T1 MRI to an MNI atlas in Freesurfer (Reuter, Rosas, & Fischl, 2010; with MNI 305 atlas; RRID:SCR_001847), and applied the resulting transformation matrix to the electrode coordinates. Brodmann areas (BA) for each electrode were identified by registering the MNI‐space electrode positions in Talairach space and labeled with the Talairach Daemon Client (Lancaster et al., 2000; RRID:SCR_000448). Monkey and sheep electrode locations were identified based on stereotaxic coordinates measured during the surgical placement of electrodes and compared to standard macaque (Saleem & Logothetis, 2012). Sheep electrode locations were estimated by landmarks (e.g., bregma) and previous somatosensory‐evoked potential studies, as MRI and stereotaxic surgical equipment were unavailable (Dinopoulos et al., 1985; Gierthmuehlen et al., 2014; Johnson et al., 1974).

#### 2.3.2 Connectivity and individual‐level statistical analysis

Pairwise connectivity across the entire resting‐state time series from each individual's recording was calculated for all electrode pairs within each individual human, monkey, and sheep. We calculated two connectivity measures: 1) amplitude‐amplitude Pearson correlation, a measure of the consistency of variation in amplitude between two signals over time, and 2) phase‐locking value (PLV; Lachaux, Rodriguez, Martinerie, & Varela, 1999), a measure of the consistency of the difference in phase between two oscillating signals over time (PLV = *Σe* ^i*Δθ^ where *Δ*θ is the instantaneous difference in phase between the signals from any two electrodes). PLV is specifically sensitive to the degree of synchronization between two signals, separately from any offset or delay between the signals. Both connectivity measures specifically quantify synchrony between regions; phase synchrony implies a degree of functional integration between regions or parts of a region (Sauseng & Klimesch, 2008).

Statistical significance for pairwise connectivity was established using a nonparametric, maximum‐value permutation test (Weaver et al., 2016). We generated surrogate data by randomly shuffling the phase, for PLV, or amplitude, for amplitude correlation, of the broadband signal, re‐extracting the frequency bands, and recalculating the connectivity measures as before. Each iteration of the permutation procedure produced (n × n)/2 – n interactions, where n is the number of electrodes for a given subject. In humans, this ranged from 64 to 108 and in monkeys from 20–35, and 32 in both sheep (Table 1). We generated a null distribution of values by retaining the top 50 highest values, and repeated the process 20 times, for a total of 1000 values in the statistical distribution. Because we retained the maximum value across all channel pairs, this approach is highly conservative, as it generates a null distribution of maximum connectivity values. We then identified the 95th percentile of this null distribution (i.e., alpha level of *p *< 0.05 of random interactions) as the threshold for retaining values as statistically significant, and discarded connectivity values below this threshold. As the method for generating the null distribution includes the entire array of signals, no further correction for multiple comparisons is needed (Casimo et al., 2016).

#### 2.3.3 Anatomical labeling and group‐level statistical analysis

**Figure 2:**
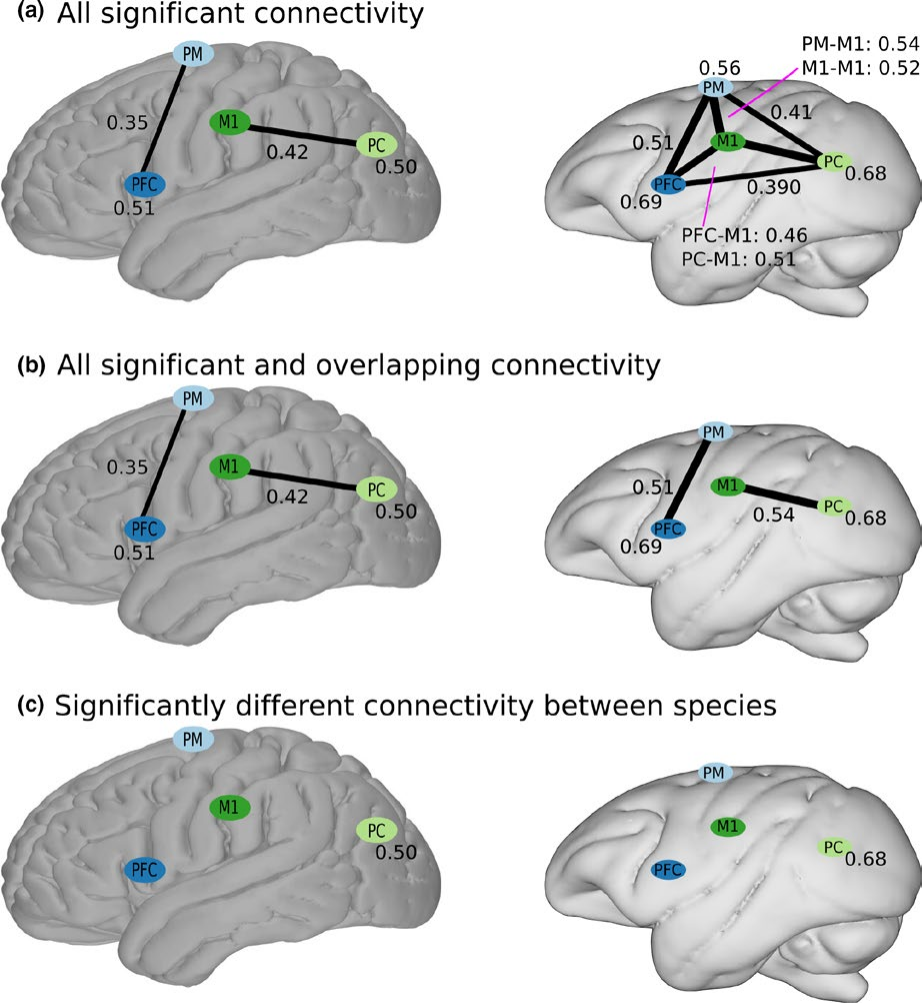
Demonstration of statistical analysis process in comparing humans and macaque monkeys. (a) for each species, connectivity values for a given region or pair of regions are pooled within an individual, filtered for statistical significance using a permutation test, and significant values are retained and pooled among all individuals. (b) connections between or within regions that had statistically significance in both species were retained. (c) connectivity values for a given connection are compared between species with a Mann–Whitney *U*‐test, and statistically significant comparisons’ values are retained. Correction for multiple comparisons was with the Bonferroni method, correcting for the number of contrasts within each frequency band for each connectivity measure

Using the electrodes’ anatomical location as identified stereotactically in surgery, we grouped the monkey electrodes into four regions: prefrontal cortex, premotor and supplementary motor cortex, primary motor cortex, and parietal cortex (Thiebaut de Schotten et al., 2012). We then identified all electrodes in the human subjects clustered in these four areas for comparison, as the anatomical extent of the monkey electrodes was more limited. Electrodes were then pooled 1) between each pair of anatomical regions and 2) within a single anatomical region (self‐pairing), and finally 3) channel‐pair labels were binned across subjects within a species (Figure 2a).

Between species, we only quantitatively compared the mean connectivity values (within or between regions) that were statistically significant in both species (Figure 2b). There are multiple reasons that a connectivity value may not be statistically significant, most notably in this case if a pair of regions have relatively few or no electrode pairs linking them, so the lack of attendant connectivity value does not imply actual lack of connectivity (Laumann et al., 2015). Consequently, we restricted further analysis to comparisons where we could determine significant connectivity values for both species.

**Figure 3:**
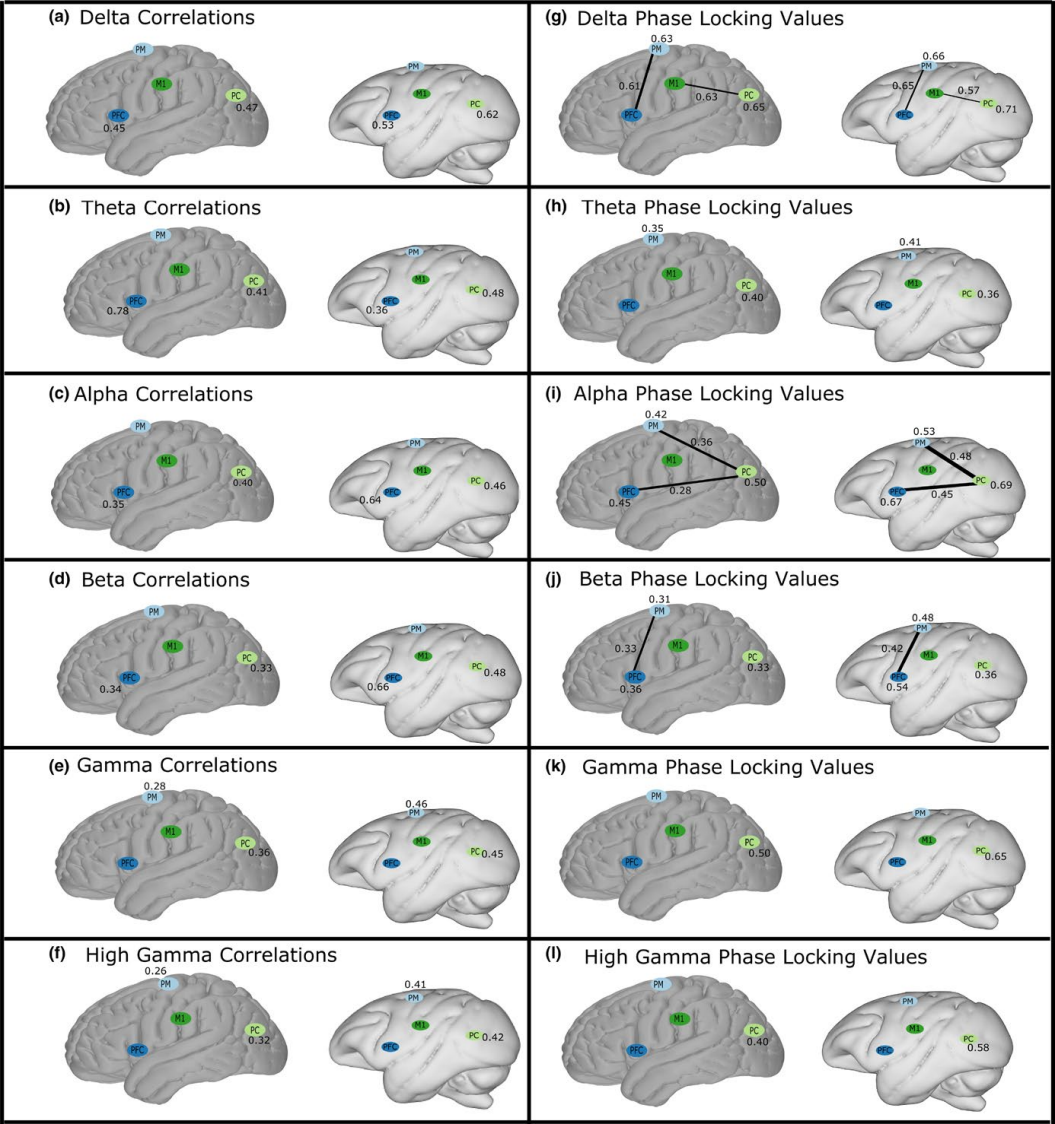
Comparison of strength of correlation (a–f) and phase locking value (g–l) in humans and macaque monkeys. Only connectivity values that were significantly different between the species are shown, as illustrated in Figure 2. Regions are prefrontal cortex (PFC), premotor cortex (PM), motor cortex (M1), and parietal cortex (PC). Connectivity values are shown for the six potential edges connecting different pairs of regions, and for within the four regions, for a total of ten possible connectivity values per connectivity measure and per frequency. Values shown next to each value or region represent connectivity strength

We performed a nonparametric Mann–Whitney *U*‐test comparing humans and monkeys for each region‐to‐region or within‐region connectivity strength. We corrected for multiple comparisons (total of ten comparisons, region‐to‐region or within‐region, per band) with the Bonferroni method within each frequency band, and connections with statistically significant differences were retained (Figure 2c). Consequently, all results and figures (Figure 3) below show connectivity between and within regions where humans and monkeys were both statistically significant separately, and were significantly different from each other.

## 3 RESULTS

### 3.1 Macaque monkeys and humans

The main focus of our approach is a comparative analysis of humans and macaque monkey connectivity estimates spanning homologous, gross cortical loci. We assessed differences in connectivity strength between the two species in 60 (four intraregion connections + six interregion connections, at six frequency bands) potential connections, per connectivity measure, in each species. Overwhelmingly, the comparative cross‐species analyses yielded similar results across frequency bands and connectivity approaches. However, we noticed several interspecies incongruities in the spatial and frequency distribution. Here we will focus on these differences.

Both connectivity measures are specifically measures of synchrony. They can be variously interpreted as the synchronized exchange of information between regions, combining information from multiple regions (such as in sensory integration), precise timing, or sequential activity (J.‐P. Lachaux, Axmacher, Mormann, Halgren, & Crone, 2012; Sauseng & Klimesch, 2008). From here forward, when we discuss “correlation strength” or “PLV strength,” we refer to the average connectivity between the pair of regions or within the region mentioned. This specifically is the mean of the connectivity values between all of the pairs of electrodes contained within or between the defined regions. Consequently, connectivity values presented represent the mean synchrony observed between all the relevant regions.

Overall, interspecies connectivity differences were overwhelmingly observed in connectivity within a single region, far more often than in connectivity between two different regions. Further, two general trends appeared: first, correlation showed relatively larger differences in magnitude between species than PLV did between species. Second, the differences between species were more widespread across cortex in PLV relative to correlation. In both measures, the species differed more often in lower frequencies than in higher frequencies. However, it is important to note that the differences were the exception rather than the rule, and correlation and PLV strengths were not different between the species in 80% and 72% of comparisons, respectively.

### 3.2 Interspecies differences between correlation and phase locking

We first examined broad trends of differences between the two connectivity measures. Of the 60 comparisons of connectivity strength between species, 12 interspecies comparisons of correlation strength (20%) were significantly different (Figure 3a–f). All these were within‐region correlations, which represent 40% of the possible connections. In contrast, 17 comparisons of PLV strength were significantly different between species (28%) (Figure 3g–l), 42% more than there were differing correlations (Figure 3a–f). Twelve of these (71%) were within‐region PLVs (Figure 3g–l). The differences between species are more widespread in phase‐based than in amplitude‐based synchrony, but the magnitude of the difference was generally larger in correlations than in PLVs.

Of the 12 interspecies differences in correlation strength, four of those connections’ PLV strengths were not different between the species (33% of differing correlations). These were observed in intra‐PFC connectivity in delta (Figure 3a,g) and theta (Figure 3b,h), and intrapremotor cortex connectivity in gamma (Figure 3e,k) and HG (Figure 3f,l).

Of the 17 interspecies differences in PLV strength, nine of those connections’ correlation strengths were not different (53% of PLV interspecies differences). These were intrapremotor cortex connectivity in delta (Figure 3a,g), alpha (Figure 3c,i), and beta (Figure 3d,j); premotor‐PFC connectivity in delta (Figure 3a,g), and beta (Figure 3d,j); M1‐parietal cortex connectivity in delta (Figure 3a,g); intrapremotor cortex connectivity in theta (Figure 3b,h); premotor‐parietal cortex connectivity in alpha (Figure 3c,i); and parietal‐PFC connectivity in alpha (Figure 3c,i). Five of these nine inconsistencies between the connectivity measures were between two different regions, and four were connectivity within a region.

### 3.3 Interspecies differences between frequencies

We also observe differences between the species in both connectivity measures in the six frequency bands. As noted, there were 71% as many interspecies comparisons with significantly different correlation strengths as significantly different PLV strengths. Consequently, knowing there were disparities between the connectivity measures, we assessed patterns in both measures in the six frequency bands.

The frequency band with the least agreement between the connectivity measures is alpha, with five significant differences within and between regions in PLV strengths (Figure 3i), and two in correlation strengths (Figure 3c), both of which were also different in PLV strength. In contrast, in both gamma and HG, there were few differences between the species: two significantly different correlation strengths between species in each frequency (Figure 3e–f), and only one PLV strength was different between species in each frequency (Figure 3k–l), which overlapped with correlation differences in both. Finally, there were just two significant differences in theta in both correlation (Figure 3b) and PLV (Figure 3h) strength, but only one overlapped in space between measures. In no frequency band did either of the two connectivity measures completely agree.

Above we summarized aggregate differences between the species across all frequencies. Now we separate the differences by frequency: in delta, 20% of correlation strengths (Figure 3a) and 40% of PLV strengths (Figure 3g) were different between the species; in theta, 20% of correlation (Figure 3b) and 20% of PLV strengths (Figure 3h); in alpha, 20% of correlation (Figure 3c) and 50% of PLV strengths (Figure 3i); in beta, 20% of correlation (Figure 3d) and 30% of PLV strength (Figure 3j); in gamma, 20% of correlation (Figure 3e) and 10% of PLV strengths (Figure 3k); and in HG, 20% of correlation (Figure 3f) and 10% of PLV strengths (Figure 3l). All but one (within parietal cortex) of these differing connection strengths were more synchronous in monkeys than in humans.

### 3.4 Interspecies differences between regions

Finally, we examine differences within regions and between pairs of regions at each frequency band and in each connectivity measure. Again, as noted above, there were very few statistically significant differences in interregional connectivity strength. For connectivity between pairs of different regions, 14% of PLV strengths were different between species, but no correlation strengths were different. In contrast, for comparisons of connectivity strength within a single region, 50% of correlation strengths and 50% of PLV strengths were different between species.

Within prefrontal cortex, we observed differences between the species in alpha (Figure 3c,i) and beta (Figure 3d,j) correlation and PLV, as well as in delta correlation (Figure 3a). Within premotor cortex, the species were significantly different in delta through beta PLV strength (Figure 3g–j), and gamma and HG correlation strength (Figure 3e–f). Within motor cortex, the species were not significantly different in either measure at any frequency (Figure 3). Within parietal cortex, all comparisons between species were significantly different (Figure 3); human connectivity was stronger than monkey connectivity only in theta PLV, the only connection more synchronous in humans (Figure 3h).

We observed the following additional connectivity patterns across the six pairs of different regions, with monkey connectivity stronger in all connections. Between PFC and premotor cortex, the species differed in delta (Figure 3g) and beta (Figure 3j) PLV. Between PFC and parietal cortex, and also between premotor and parietal cortex, the species differed only in alpha PLV strength (Figure 3i). Finally, between motor and parietal cortex, the species differed in only delta PLV (Figure 3g). Between PFC and motor cortex, and between premotor and motor cortex, there were no significant differences in connectivity between the species in either measure at any frequency (Figure 3).

As noted above, of the 29 connections in both measures that were significantly different between the species, just one (theta PLV strength within parietal cortex) was stronger in humans.

### 3.5 Interspecies differences between humans and sheep

**Figure 4:**
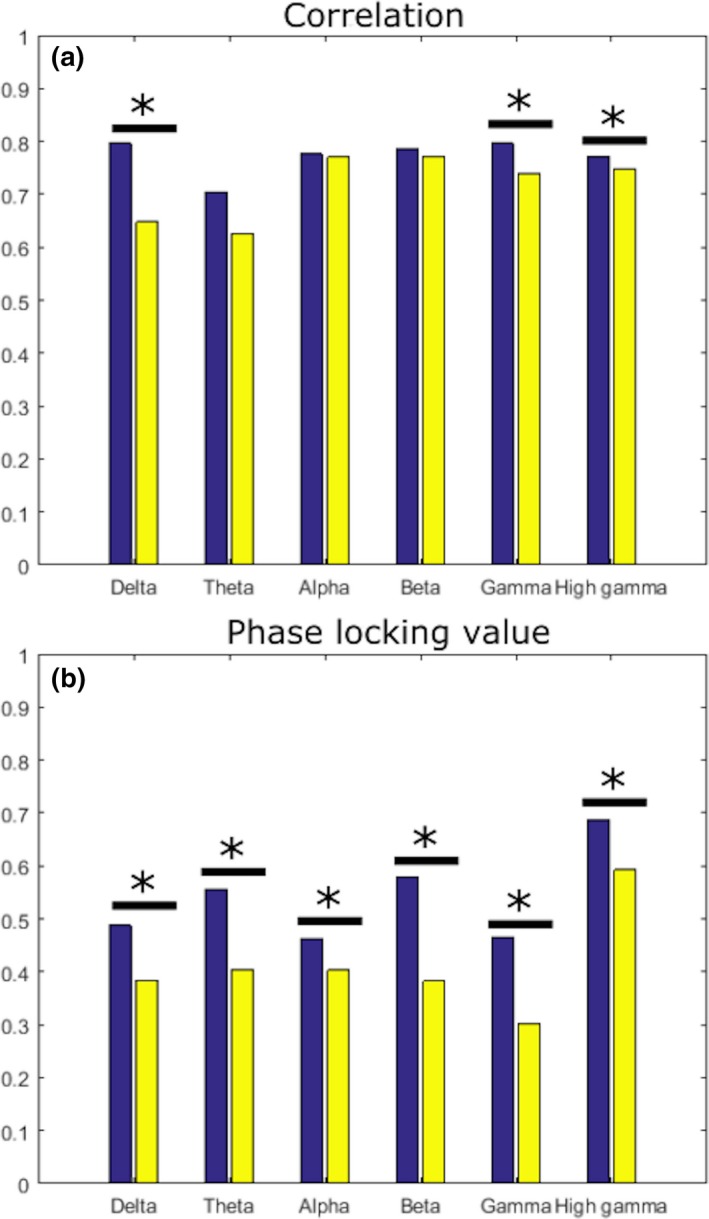
Comparison of strength of correlation (a) and phase locking value (b) between the same four humans used above (blue bars) and two sheep (yellow bars). Connectivity strength between species is compared with a Mann–Whitney *U*‐test, and statistically significant differences are marked with a star (*p* < 0.05, Bonferroni corrected)

We also assessed intrasomatosensory (parietal) cortex connectivity in two sheep. As noted above, human and monkey connectivity within parietal cortex was significantly different in all contrasts but delta correlation (Figure 3). Where human and monkey intraparietal (somatosensory) connectivity was significantly different, connectivity was stronger in monkeys in all except theta PLV, the only instance where human connectivity was stronger than monkey connectivity (Figure 3h). In contrast, sheep intrasomatosensory correlation in delta, gamma, and high gamma, and PLV in all frequencies were significantly lower than in humans (Figure 4a,b). In no frequency, in either correlation or PLV, was sheep intrasomatosensory connectivity stronger than human connectivity (Figure 4a,b).

## 4 DISCUSSION

We compared resting‐state connectivity using subdural electrophysiological methods within and between cortical regions, focusing on humans and macaque monkeys, and between humans and sheep. We observe a general pattern of functional connectivity in a behaviorally unstructured state that was generally similar between different regions when comparing human and macaque monkeys, with greater discrepancies occurring within homologous regions than between regions.

We speculate that reduced local synchrony in humans relative to monkeys may reflect the balance between heterogeneity and integration in function within and between cortical regions. Though we cannot rule out contributions from the differing electrode diameters, the similarity in the two species in connectivity between pairs of different regions implies that the increased heterogeneity in humans is local, and that functional interactions between different regions are largely preserved.

Among the differences between species, the greatest disparity was within‐parietal cortex connectivity, which differed between humans and macaques in every frequency band in both connectivity measures (Table 1, Figure 3). Within‐prefrontal cortex connectivity also differed in multiple frequency bands in both connectivity measures (Figure 3). In the minority of instances in which the species differ, human synchrony may be generally weaker than monkey connectivity potentially because of an increased functional granularity of functional parcellation or more fine‐grained regional specialization in human brain (Saleem et al., 2014; Thiebaut de Schotten et al., 2012). The prefrontal cortex is notably larger and more cytoarchitecturally complex in humans than in other primate species (Buckner & Krienen, 2013; Thiebaut de Schotten et al., 2012). This might result in greater functional heterogeneity in the neuronal populations detected by each electrode, leading to decreased synchrony and therefore connectivity strength.

This conclusion is further supported by the observation that no differences between the species were identified in within‐ primary motor cortex connectivity, one of the most evolutionarily old and well‐conserved parcels of neocortex (Arendt, Tosches, & Marlow, 2015). In sheep and human parietal cortex, connectivity patterns also differed, as they did in human and monkey within‐parietal comparisons, but human connectivity was stronger than sheep. Relative connectivity strength may be attributable to the number of different afferent sources to that cortical parcel, level of functional integration within the region, or other sources of heterogeneity.

### 4.1 Differences between connectivity measures

Collectively, the humans and monkeys are more similar in resting‐state connectivity patterns between different regions than within a single region. Furthermore, sheep and human connectivity differences, evaluated only within one region, were dissimilar in both connectivity measures much as humans and monkey values were for a single region.

The two connectivity measures we evaluated, amplitude‐amplitude correlation and PLV, were generally similar, with the bulk of the conflict between the two measures in monkey‐human comparison appearing in between‐region connectivity. This may be a result of the fact that signal phase becomes more desynchronized with respect to amplitude over greater distances (i.e., between regions) (von Stein & Sarnthein, 2000). Other potential factors that may account for these differences include that PLV is simply more sensitive to smaller inconsistencies in phase difference than correlation is to small variation in amplitude, or that differences between species in the number of electrodes in any given region.

### 4.2 Differences between regions of the brain

The vast majority of comparisons of regions’ connectivity in humans and macaques are not significantly different. This is consistent with prior fMRI comparisons between finding extensive homology between human and monkey structural and functional connectivity (Goulas et al., 2014; Hutchison & Everling, 2012; Hutchison et al., 2012; Margulies et al., 2009; Zhang et al., 2010). Our findings in electrophysiology concur with and support these fMRI observations, as the species’ connectivity patterns, based on phase and amplitude interactions across a wide range of frequency bands, are generally similar.

The differences between species were overwhelmingly concentrated in within‐region connectivity, rather than between regions. This suggests that specific local synchrony differs more substantively than long‐distance, interregional functional connectivity. However, both amplitude‐amplitude coupling and PLV are linear measures of interaction, and are not sensitive to cross‐frequency interactions. Cross‐frequency coupling (CFC) is now believed to underlie long‐distance cortico‐cortical entrainment to a greater degree possibly as a neurobiological mechanism facilitating information transfer (Canolty & Knight, 2010; Weaver et al., 2016). Further research using CFC may reveal features that are not accessible in linear measures.

We speculate that the strong similarities between species in motor cortex connectivity may indicate conserved function of the motor systems between humans and macaque monkeys, extending to multiple motor‐related areas that are functionally homologous between these two species. This is consistent with prior work using tract‐tracing methodology (Hackett et al., 2001; Thiebaut de Schotten et al., 2012).

In contrast, both within‐premotor and within‐parietal cortex connectivity was markedly different between the species across multiple frequency bands (Figure 3). For within‐premotor cortex, this included motor‐linked beta, potentially indicating localized differences in the functional architecture relating to motor planning behavior. For instance, humans may have a more complex or extensive motor repertoire than macaques, or there may be greater complexity in motor decision‐making processes. Connectivity within parietal cortex had the most pervasive differences between the species, with statistically significant differences in all frequency bands and both connectivity measures. Human parietal association areas may incorporate more afferents, more sophisticated sensory integration, or more sensory processing function relative to monkeys.

### 4.3 Contributions from sheep recordings

We found the most pervasive disparity between humans and macaque monkeys was in within‐parietal cortex connectivity (Figure 3). Our findings in sheep extend this result. The equivalent comparison of parietal cortex between humans and sheep differed in delta, theta, alpha, and beta frequencies in both connectivity measures (Figure 4). However, in contrast to the human‐monkey comparisons where monkey connectivity was almost universally stronger, human connectivity in both measures was always stronger than in sheep. We suggest that low synchrony in sheep may be indicative of weaker sensory integration than in humans, as this observation specifically pertains to parietal cortex. We are not able to draw conclusions about overall sheep connectivity patterns due to lack of coverage. However, our findings indicate that sheep are suitable for investigations of connectivity, such as for stimulation‐induced connectivity changes, that then can be translated to primates or humans for further investigation.

### 4.4 Limitations

The primary limitation of this study derives from the differing sensor properties used across the three species. Although all recordings were made with subdural electrodes, the precise composition and size of the electrodes and their spacing varied. In particular, the monkey and sheep electrodes were smaller than the humans’, and thus each electrode samples from fewer neurons. We excluded the depth electrodes also present in two of the monkeys; a comparison of human and monkey depth electrode recordings would extend our findings.

This study, as with all ECoG studies in humans, and some degree in animals, was further limited by the spatial placement and coverage of electrodes. The macaque monkey and sheep electrode placements were determined by the needs of the other studies. The human electrode placement was determined by their clinical needs, though we were able to select human subjects whose coverage generally overlapped with that of the monkeys. We suggest further research include additional subjects, human and animal, to expand spatial coverage.

In addition, the resting state is not as well defined in animals with respect to human subjects. We utilized video of animal behavior to distinguish periods of inactivity from activity. However, there is less control over animal models when ensuring complete adherence to a true resting state. In sheep, we were only able to obtain 3–4‐min‐long recordings, in contrast to 10 min in humans and monkeys. This, as well as the limited spatial extent of the sheep electrode placement, leaves opportunity for further investigation.

### 4.5 Conclusions and further studies

In conclusion, we find that human and monkey electrophysiological functional connectivity in the resting state is largely similar, particularly in interregional connectivity. The minority of significant differences occur mostly in local, intraregional connectivity, again consistent with prior studies finding consistency between humans and nonhuman primates in interregional connectivity (Hutchison et al., 2012; Hutchison, Womelsdorf, Gati, et al., 2013; Margulies et al., 2009; Zhang et al., 2010). Furthermore, in nearly all of the disparate regions, the strength of connectivity was greater in monkeys. The similarity in synchrony noted, especially between humans and monkeys, suggests a physiological link between the species that bridges the cognitive and behavioral gap.

While we cannot rule out possible differences in sensor characteristics, the variation in synchrony levels may reflect differences in the degree of functional segregation between species. We were not able to capture fMRI data in this investigation, but these results are consistent with those of extensive previous imaging‐based investigations (Hutchison et al., 2012; Hutchison, Womelsdorf, Allen, et al., 2013; Margulies et al., 2009; Zhang et al., 2010).

We suggest that this similarity supports the application of PLV and amplitude correlation in monkeys as one means of indirectly probing the architecture of neural circuits in humans. If further validated, comparability between species can enable findings from an animal model such as a monkey or a sheep, where more invasive electrophysiology (e.g., tracing, invasive implantations, single‐unit recordings) are readily available, to be used to supplement electrophysiological or imaging techniques usable in humans. This may be applicable in both studies of the origins of resting‐state connectivity or of other task‐related connectivity.

While sheep and humans differ more sharply than monkeys and humans do, an understanding of sheep connectivity properties aids in the use of sheep to study basic principles of connectivity, particularly in instances such as cortical stimulation where the ability to do highly invasive studies in sheep can provide guidance for further studies. Experimental outcomes in sheep can then be validated in other species, as we have done here. Sheep are also popular model for testing a variety of neurological diseases including stroke (Wells et al., 2012), Parkinson's disease (Baskin, Browning, Widmayer, Zhu, & Grossman, 1994; Hammock et al., 1989), and focal epilepsy (Opdam et al., 2002), and our findings potentially contribute to the cross‐species validation of these disease models.

A significant degree of cross‐frequency coupling has been observed in resting state in both humans (Weaver et al., 2016) and monkeys (Schroeder & Lakatos, 2009); differences in this phenomenon may also related to the single‐frequency effects observed here. Further work should evaluate cross‐frequency coupling (including phase‐amplitude coupling or other measures) to interrogate this possibility.

## CONFLICTS OF INTEREST

The authors have no conflicts of interest to declare.

## AUTHORS’ CONTRIBUTIONS

KC and JGO participated in study concept and design. KC, SZ, CAG, KEW, TB, EF, and JGO involved in acquisition of data. KC, LL, SZ, CAG, KEW, and JGO performed analysis and interpretation of data. KC and LL involved in statistical analysis, drafting of the manuscript, and also contributed equally to this work. TB, EF, and JGO involved in study supervision and also obtained funding for the manuscript.
